# Dietary patterns among children and adolescents in Germany consuming vegetarian, vegan or omnivore diets: results of the VeChi Youth Study

**DOI:** 10.1007/s00394-024-03497-6

**Published:** 2024-09-23

**Authors:** Lea Hansch, Morwenna Fischer, Alfred Längler, Andreas Michalsen, Stine Weder, Markus Keller, Ute Alexy, Ines Perrar

**Affiliations:** 1 Department of Nutritional Epidemiology, Institute of Nutritional and Food Science (IEL), University of Bonn, DONALD Study, Heinstück 11, 44225 Dortmund, Germany; 2 Faculty of Human Resources, Health and Social Work, University of Applied Sciences (FHM), 33602 Bielefeld, Germany; 3 Department of Pediatrics, Gemeinschaftskrankenhaus, Herdecke, Germany; 4 Faculty of Health, Professorship for Integrativ Pediatrics, Witten Herdecke University, Witten, Germany; 5 Institute of Social Medicine, Epidemiology and Health Economics, Charité- Universitätsmedizin Berlin, 10117 Berlin, Germany; 6 Research Institute of Plant-Based Nutrition, 35444 Gießen/Biebertal, Germany

**Keywords:** Vegetarian diet, Vegan diet, Children, Adolescents, Dietary patterns, Nutrient intake

## Abstract

**Purpose:**

To identify dietary patterns of vegetarian, vegan and omnivore children and adolescents in Germany and to examine associations with nutrient intake.

**Methods:**

First, three principal component analyses based on 17–22 food groups were used to identify dietary patterns among vegetarians (*n* = 145, 3-day weighed dietary records), vegans (*n* = 110) and omnivores (*n* = 135) from the cross-sectional Vegetarian and Vegan Children and Youth (VeChi Youth) Study (2017–2019, 6–18 years, 57% girls). Secondly, these patterns were correlated (Spearman correlations) with energy and nutrient intakes.

**Results:**

Among vegetarians, 3 dietary patterns were identified (“Animal foods”, “Vegetables and fruits”, “Meat alternatives and potatoes”) accounting for 32.7% of the variance in food group intake. In the vegan group, 4 patterns were identified (“Vegetables and legumes”, “Refined carbohydrates”, “Meat alternatives and juices”, “Fruits and convenience foods”) accounting for 43.2% of the variance. Among omnivores, 5 (“Flexitarian”, “Vegetables and fruits”, “Dairy Products”, “meat and convenience foods”, “Refined grains and juices”) patterns accounting for 43.0% of the variance could be identified. Regardless of diet group, both more favorable dietary patterns (“Vegetables and fruits”, “Meat alternatives and potatoes”, “Vegetables and legumes”, “Fruits and convenience food”, “Flexitarian”) correlated with higher micronutrient density and less favorable dietary patterns (“Animal foods”, “Refined carbohydrates”, “Meat alternatives and juices”, “Dairy products”, “Meat and convenience food”, “Refined grains and juices”) with unfavorable nutrient profiles were found.

**Conclusion:**

Various dietary patterns exist within omnivore, vegetarian, and vegan diets of children and adolescents, which differ in their contribution to nutrient intake. It is therefore necessary to distinguish between different dietary patterns, also within the vegetarian and vegan diet.

**Supplementary Information:**

The online version contains supplementary material available at 10.1007/s00394-024-03497-6.

## Introduction

Vegetarian (excluding meat and fish/seafood) and vegan (excluding all foods of animal origin) diets are becoming increasingly popular [1, 2], also among children and adolescents. In a representative German survey, the proportion of vegetarian adolescents (12–17 years old) increased from 1.6% (2006) to 5.0% (2015–2017) [3]. Plant-based diets, such as vegetarian or vegan diets, are associated with various health benefits, particularly regarding the prevention of cardiovascular diseases [4–10]. Compared to an omnivore diet, a more favorable dietary fatty acid profile was found in vegans (lower intake of saturated fatty acids, higher intake of polyunsaturated fatty acids) as well as a higher intake of dietary fiber, vitamin C, vitamin E, folate, magnesium and iron [11, 12]. However, limiting food choices by excluding foods of animal origin is also discussed in terms of increased risk of certain nutrient deficiencies, in particular in the pediatric population [13, 14]. The growth and development processes during this vulnerable phase of life are associated with an increased energy and nutrient requirement in relation to body weight. Therefore, an adequate nutrient intake during childhood and adolescence is of particular importance for normal physical and mental development [15–17]. Potentially critical nutrients in a vegetarian child diet are iodine and vitamin D, just as in omnivore diets, as well as iron, zinc, and long-chain omega-3 fatty acids. In vegan diets, in addition to the same critical nutrients for vegetarian diets, the intake of protein, vitamin B_12_, vitamin B_2_ and calcium also need attention [18]. To ensure an adequate intake of these nutrients careful planning is required with a special focus on nutrient-dense foods, such as whole grains, legumes, nuts and seeds, and on fortified foods [18, 19]. Particularly in vegan diets, attention should also be paid to a targeted intake of fortified foods [19].

One way to describe the overall diet of a population in its complexity is through dietary pattern analysis [20]. Exploratory, *a posteriori*, pattern analyses are statistical methods that generate population-specific dietary patterns from the collected dietary intake data of a population [21]. A number of studies have investigated the exploratory dietary patterns of children and adolescents in Western countries [22–37]. In these studies, different dietary patterns could be identified, which not only showed significant differences in the quality of food choices, but were also associated to different extents with energy and nutrient intake [22, 25, 30, 37]. Among adult vegans, it has been shown that in addition to traditional vegan dietary patterns focusing on unprocessed foods, there are also unhealthier dietary patterns characterized by higher intake of processed foods, i.e., vegan sweets, snacks, fast food, and convenience meals [38, 39]. To the best of our knowledge, there are no studies on dietary patterns among vegetarian or vegan children and adolescents and potential associations with nutrient intake during this vulnerable phase of life. Thus, the aim of the present study was to identify dietary patterns within the vegetarian, vegan, and omnivore diets of children and adolescents using data from the Vegetarian and Vegan Children and Youth Study (VeChi Youth Study). In addition, we explored the correlations between the extracted dietary patterns and nutrient intake.

## Methods

### The VeChi Youth Study

The VeChi Youth Study is a cross-sectional study that collected data on diet, lifestyle, anthropometry, and nutritional status of vegetarian, vegan, and omnivore children and adolescents in Germany. All examinations were performed with parental and older participants’ (≥ 14 years) written consent. Examinations included a 3-day weighed dietary record, anthropometric measurements, a spot urine, a blood sample as well as questionnaires on socio-demographic factors and lifestyle. Examinations and interviews were performed by trained staff. Further details about the study have been described elsewhere [40–42].

The VeChi Youth Study was conducted according to the guidelines of the Declaration of Helsinki and approved by the Ethics Committee of Witten-Herdecke University (139/2017). The study has been registered at the German Clinical Trials Register (DRKS00012835).

### Study sample

The VeChi Youth Study included 401 healthy children and adolescents aged 6–18 years who were examined in three study centers in Germany. One participant age 5.5 years and one participant age 19.1 years were also included into the study sample. Due to the low prevalence of vegans in Germany [2, 3], the collective had to be recruited specifically. Exclusion criteria were as follows: (a) diagnosed diseases that could affect the studied variables (e.g., enteropathy, pancreatic diseases, metabolic disorders like phenylketonuria or fructose malabsorption), and (b) special diets other than vegan or vegetarian diet, e.g., predominantly (70%) raw food diet (according to [43]). Of the 401 participants included, 390 (girls *n* = 221; 57%) compleed a three-day weighed food record. Accordingly, data from 110 vegans (28%), 145 vegetarians (37%) and 135 omnivores (35%) were available for the present analysis (Supplementary Fig. 1). The power calculation for the VeChi Youth Study was carried out for serum ferritin as the primary outcome, described in detail elsewhere [41].

### Questionnaires

An online questionnaire was used to record socio-demographic variables, such as nationality, monthly income, or parental education, as well as nutritional variables, such as duration of vegetarian or vegan diet, motivation for the diet or intake of dietary supplements. The Winkler Index was used to describe socioeconomic status [44]. This index is made up of three scores on school education, training, and net household income (1–7 points each). If the mother’s and father’s scores differed, the higher score was used and categorized as the family index for socioeconomic status. Using the Winkler index, the families were divided into three categories: low (3–8 points), medium (9–14 points) or high (15–21 points) social status. Physical activity was assessed using the validated Adolescent Physical Activity Recall Questionnaire [45]. The duration of physical activity (hours/week) and MET minutes per week were calculated using the information provided on organized (e.g. training in a sports club) or unorganized sports activities (recreational sports, e.g. football matches with friends or jogging) and using databases on the metabolic intensity of the activities [46, 47].

### Anthropometric measurements

Body weight (kg) was measured on the day of the examination in underwear and without shoes using an electronic column scale (Seca 799, graduation 100 g, up to 150 kg body weight). Height (cm) was determined using a stadiometer (Seca 222, graduation 1 mm). The body mass index (BMI, kg/m^2^) was calculated from the quotient of body weight (kg) and the squared height (m). The gender- and age-independent BMI standard deviation scores (BMI-SDS) were calculated using the national reference data from Kromeyer-Hauschild [48]. The BMI-SDS indicates how many standard deviations an individual BMI is above or below the BMI median of the reference population for a given age and gender.

### Dietary assessment

Dietary intake of study participants was assessed using 3-day weighed dietary records as described in detail elsewhere [40]. In short, over a period of three consecutive, freely selectable days, participants and/or their parents weighed and recorded all foods and beverages consumed as well as leftovers using electronic scales. If weighing was not possible (e.g., in case of eating out) participants were asked for semi-quantitative recording using household measures (e.g., tablespoons, pieces). For commercial food products (e.g., ready-to-eat meals and meat or dairy alternatives), the exact brand name was also recorded. Missing data were assessed by the study staff by requesting the information from the parents via email.

Energy, nutrient and food group intakes were calculated using the LEBTAB food composition database (Version III, DONALD Study, University of Bonn) [49] which contains nutritional information on staple foods based on standard German food composition tables (Bundeslebensmittelschlüssel BLS 3.02) as well as brand-specific foods and dietary supplements. The energy and nutrient composition of commercial food products were calculated by recipe simulation based on the nutrient and ingredient declaration.

To calculate food group intakes, each food item consumed was assigned to one of 22 food groups (Supplementary Table 1). Food groups were formed based on similarities in nutrient profile, ingredients, or culinary use. Mean intake of each food group (in g/day) was determined by summing daily intake amounts and then averaging, i.e., dividing by the 3 dietary record days. Food group intake was calculated as consumption amount per 1000 kcal total energy intake per day (g/1000 kcal/day) to account for differences in energy requirements and total quantitative intake per day between boys and girls as well within the wide age range of the study population [40]. For dietary pattern analysis, food group intakes (g/1000 kcal) were standardized (mean 0, standard deviation 1) using z-transformation.

### Diet group classification

Vegetarian, vegan or omnivore diets were categorized according to the following question during recruitment:

Do you/does your child eat.

- a vegetarian diet (no meat, sausage, fish, but dairy and/or eggs)
- a vegan diet (no meat, sausage, fish, dairy and eggs)
- an omnivore diet (including meat and/or fish)?

In addition, in the online questionnaire some crosscheck questions were asked, whether there are exceptions in food intake (e.g., occasional intake of dairy products in vegan diets or occasional intake of meat or fish/seafood in vegetarian diets). Based on these control questions, 24 study participants were reclassified [40].

### Statistical analysis

All statistical analyses were performed using SAS^®^ 9.4. The significance level was set at *P* < 0.05. Data were checked for plausibility and outliers.

A separate principal component analysis (PCA) was conducted for each diet group (vegetarian, vegan, omnivore). The suitability of the data for PCA was assed using the Kaiser-Meyer-Olkin (KMO) and Bartlett’s sphericity test. With values < 0.5, the KMO test indicated insufficient data adequacy (vegetarian group: 0.39, vegan group: 0.18, omnivore group: 0.31) whereas Bartlett’s test confirmed the data suitability for PCA (all diet groups *P* < 0.0001).

Due to the exclusion of animal source food groups in the vegetarian and vegan diets, the number of food groups analyzed differed depending on the diet group (omnivore: 22 food groups, vegetarian: 21 food groups, vegan group: 17 food groups).

Principal component analyses (SAS^®^ procedure PROC FACTOR (method = prin)) were performed using the following criteria to identify the number of significant dietary patterns [50]: Eigenvalue > 1; Scree plot evaluation (identification of a “break” in the graphical presentation of eigenvalues; Interpretability (adequate number of food groups with high factor loadings in the dietary pattern: according to Hatcher [51], at least 3 factor loadings ≥ |0.4|). The components were rotated using orthogonal transformation (varimax rotation, SAS^®^ option rotate = varimax) to obtain uncorrelated patterns with a simpler structure and better interpretability. The number of patterns to be extracted was selected using the “nfact” option [50].

Food groups with factor loadings ≥ |0.2| were considered to contribute significantly to a dietary pattern [31, 33, 52] and were therefore used for the description of dietary patterns. Factor loadings ≥ |0.4| were considered high. The identified dietary patterns were labeled according to the food groups with high positive factor loadings or qualitative characteristics of the diet. Although the chosen labels do not comprehensively describe the underlying dietary patterns, they facilitate the presentation and discussion of the results.

Each study participant was assigned a score for each dietary pattern, which was the sum of the individual standardized food group intakes (g/1000 kcal) weighted by the corresponding factor loadings of the food groups in each dietary pattern. These pattern scores indicate the degree of adherence to the respective dietary pattern. Thus, a high individual pattern score indicates that the diet of the study participant strongly matches the dietary pattern under consideration.

Spearman correlation coefficients were calculated to examine the association between dietary pattern scores and energy and nutrient intakes within the diet groups. A positive correlation between a dietary pattern and a nutrient indicates that a high dietary pattern score (high correspondence between a participant’s diet and this pattern) is associated with a higher nutrient intake. A negative correlation, on the other hand, means that a high score in the pattern is associated with a lower nutrient intake. Because nutrient intake is strongly correlated with energy intake, the nutrient density approach was used to adjust for energy intake [53]. For this purpose, macronutrients were analyzed as %E and micronutrients and dietary fiber as intakes per 1000 kcal [53, 54]. According to Cohen’s established categorization [55] and in line with a previous study on correlations between children’s dietary patterns and nutrient intake [37], |r| > 0.5 was interpreted as a strong correlation.

## Results

### Sample characteristics

The characteristics of the study population, stratified by diet group, are shown in Table 1. The median age of the study population was 12.5 years and age ranged from 5.5 to 19.1 years. The median BMI-SDS was below zero in all diet groups. Most of the participants (*n* = 278; 71.3%) were from families with a high socioeconomic status. Vegetarian subjects stated that they had been following this diet for a median of 4.1 years and vegan subjects for a median of 3.4 years. In all three diet groups, the participants completed the 3-day weighed dietary records predominantly on two weekdays as well as one weekend day and only minor differences were observed regarding the seasons in which the records were completed (Supplementary Table 2). Further characteristics of the study population and data on food group intake have been described in detail elsewhere [41, 42].

**Table 1:** Sample characteristics of VeChi Youth Study participants (*n* = 390, 6–18 years old) stratified by diet group

	Diet group			
	Vegetarian (*n* = 145)	Vegan (*n* = 110)	Omnivore (*n* = 135)	*P* ^*1*^
Girls	87 (60.0)	73 (66.4)	61 (45.2)	
Boys	58 (40.0)	37 (33.6)	74 (54.8)	
Age (years)	12.4 (9.2; 16.0)	12.8 (9.0; 16.9)	12.3 (9.5; 16.2)	0.8741
Height (cm)	157.2 (136.3; 169.7)	155.5 (132.2; 166.0)	159.9 (138.0; 173.2)	0.2182
Weight (kg)	44.5 (29.8; 58.2)	43.6 (27.7; 55.7)	42.8 (31.3; 59.8)	0.2259
BMI-SDS	–0.4 (–0.93; 0.17)	–0.6 (–1.1; 0.12)	–0.2 (–0.9; 0.4)	0.0242
Duration of dietary regimen (years) ^2^	4.1 (2.1; 6.6)	3.4 (1.8; 6.5)	n.a.	0.1084
Main motive for diet ^3^				
Ethics	96 (69.6)	75 (68.8)	n.a.	
Parents	19 (13.8)	14 (12.8)	n.a.	
Health	3 (2.2)	12 (11.0)	n.a.	0.0100
Ecology	10 (7.3)	5 (4.6)	n.a.	
Other	10 (7.3)	3 (2.8)	n.a.	
Socioeconomic status ^4^				
High	105 (75.0)	67 (62.0)	106 (80.9)	
Middle	32 (22.9)	37 (34.3)	25 (19.1)	0.0155
Low	3 (2.1)	4 (3.7)	0 (0.0)	
Smoking in the household (never) ^5^	135 (96.4)	102 (93.6)	128 (97.7)	0.3563
Physical activity ^6^				
(hours/week)	2.7 (1.8; 4.0)	2.9 (1.6; 4.3)	3.0 (2.0; 4.1)	0.6324
(MET-minutes/ week) ^6^	1120.5 (671.3; 1629.0)	985.5 (630.0; 1662.0)	1160.3 (753.5; 1751.0)	0.4578

Values are n (%) or median (Q1; Q3)

BMI-SDS = standard deviation score of body mass index; MET, metabolic equivalent of task

^1^ Chi2-Test or Fisher’s exact test (categorical variables) or Kruskal Wallis test or T-Test (continuous variables) or ANCOVA adjusted for age and sex (SDS-BMI, TEI), P-values adjusted for multiple testing according to the False Discovery Rate method (see also [42])

^2^ Duration of dietary regimen: 11 missings (vegetarian: *n* 8; vegan: *n* 3)

^3^ Main motive for diet: 8 missings (vegetarian: *n* 7; vegan: *n* 1)

^4^ Socioeconomic status: High social class: Winkler index > 14, middle social class: Winkler index > 9 to 14, low social class (Winkler index ≤ 9), 11 missings (vegetarian: *n* 5, vegan: *n* 2; omnivore: *n* 4)

^5^ Smoking in the household: 10 missings (vegetarian: n 5; vegan: n 1, omnivore: n 4)

^6^ Physical activity: 1 missing (only vegan)

### Dietary patterns

**Fig. 1:**
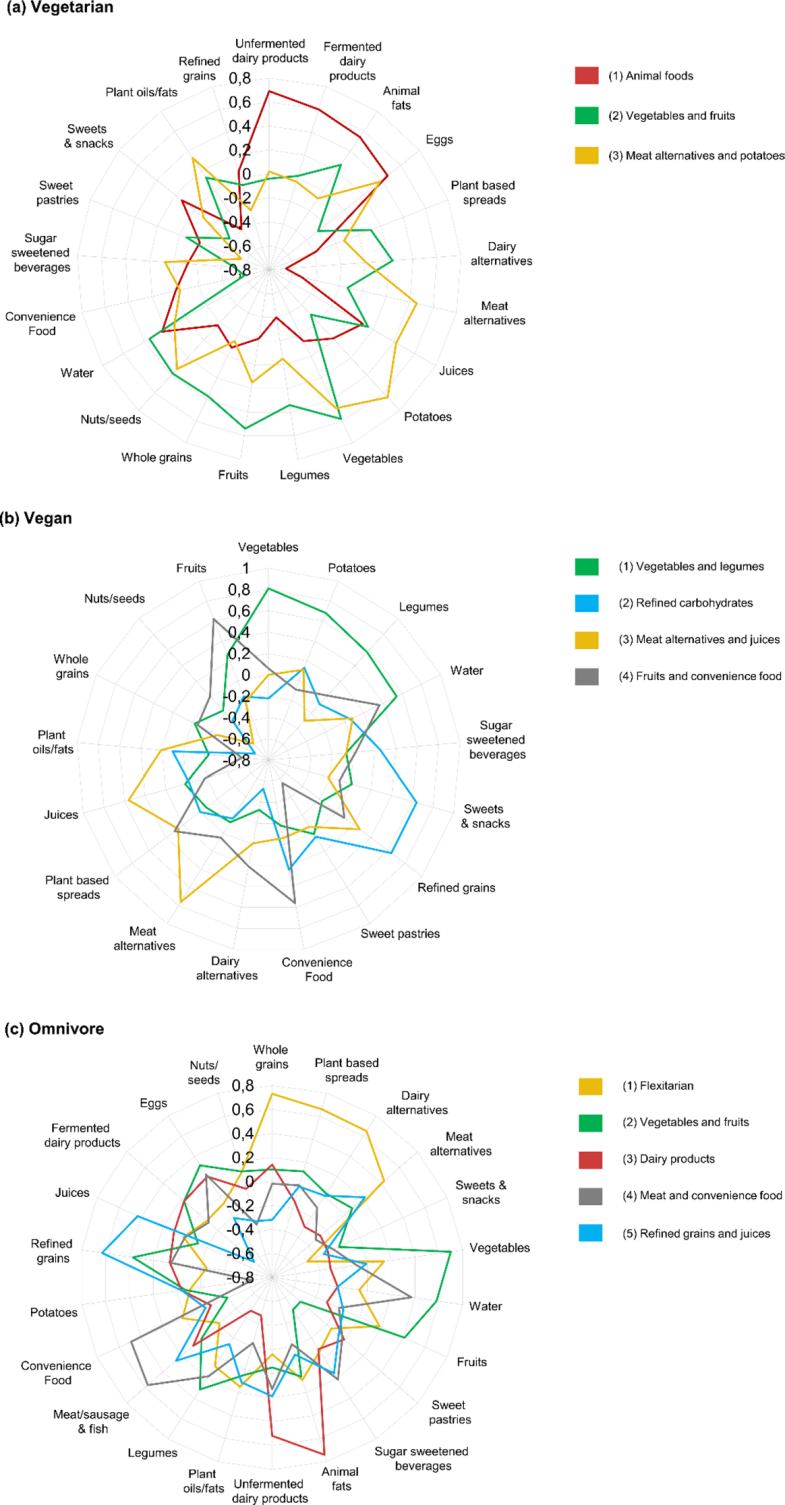
Spider diagram of factor loadings for the dietary patterns identified by principal components analysis among (**a**) vegetarian, (**b**) vegan, and (**c**) omnivore children and adolescents in the German VeChi Youth Study

Three predominant components (dietary patterns) were identified in the vegetarian diet group (Supplementary Table 3), four in the vegan group (Supplementary Table 4), and five in the omnivore group (Supplementary Table 5). The identified components together explained 32.7% of the variance in food group intake in the vegetarian group, 43.2% in the vegan group, and 43.0% in the omnivore group. The dietary patterns and factor loadings are summarized in Fig. 1 and described in detail below.

### Vegetarian diet group

The first identified dietary pattern “Animal foods” was characterized by high positive factor loadings for unfermented and fermented dairy products, animal fats, and eggs or egg dishes. In contrast, high negative factor loadings were found for plant-based dairy and meat alternatives and for legumes. The second component “Vegetables and fruits” had high positive factor loadings for vegetables and fruits as well as high negative factor loadings for convenience foods and sugar-sweetened beverages. The third pattern “Meat alternatives and potatoes” was associated with high positive factor loadings for potatoes and potato products, vegetables, plant-based meat alternatives, and juices and had a high negative factor loading for sweet pastries.

### Vegan diet group

The first identified pattern was labeled “Vegetables and legumes” because it had high positive factor loadings for vegetables, potatoes and potato products, legumes, and water. The second component “Refined carbohydrates” was characterized by high positive factor loadings for sweets and salty snack foods and refined grains and had high negative factor loadings for whole grains and plant-based dairy alternatives. The third component “Meat alternatives and juices” was associated with high positive factor loadings for plant-based meat alternatives and juices and high negative factor loadings for nuts and seeds. The fourth pattern “Fruits and convenience foods” had high positive factor loadings for fruits and convenience foods as well as high negative factor loadings for sweet pastries and vegetable oils and fats.

### Omnivore diet group

The first component called “Flexitarian” in the omnivore diet group was characterized by high positive factor loadings for whole grains, plant-based spreads, dairy alternatives, and meat alternatives, and had high negative factor loadings for sweets and salty snack foods. The second component “Vegetables and fruits” had high positive factor loadings for vegetables, water, and fruits and high negative factor loadings for sweet pastries and sugar-sweetened beverages. The third component “Dairy Products” had high positive factor loadings for animal fats and unfermented dairy products. High negative factor loadings were also found for vegetable oils and fats and legumes in this pattern. The fourth component “meat and convenience foods” in the omnivore group was associated with high positive factor loadings for meat and fish as well as convenience foods and with high negative factor loadings for potatoes and potato products. The fifth dietary pattern “Refined grains and juices” was characterized by high positive factor loadings for refined grains and juices. A high negative factor loading was found in this pattern for fermented dairy products.

### Association between dietary patterns and energy and nutrient intake

For each dietary pattern, several significant associations were found with energy and nutrient intakes (Tables 2, 3 and 4), but correlation coefficients |r| were often < 0.5.

**Table 2:** Spearman correlation between dietary pattern scores and energy and nutrient density in vegetarian participants of the German VeChi Youth Study (*n* = 145)

	Animal foods	Vegetables and fruits	Meat alternatives and potatoes
Energy (kcal/100 g)	-0.12	-0.32***	-0.37***
Fat (%E)	0.24**	-0.09	0.08
SFA (%E)	0.60***	-0.13	-0.30***
MUFA (%E)	0.15	-0.12	0.18*
PUFA (%E)	-0.54***	0.15	0.40***
Linoleic acid (%E)	-0.57***	0.14	0.36***
α-Linolenic acid (%E)	-0.22**	-0.01	0.32***
Protein (%E)	0.18*	0.03	0.10
Carbohydrates (%E)	-0.29***	0.09	-0.13
Added sugars (%E)	0.15	-0.46***	-0.27**
Dietary fiber (g/1000 kcal)	-0.65***	0.69***	0.28***
Retinol-Equivalents (µg/1000 kcal)	0.01	0.34***	0.28***
Tocopherol-Equivalents (µg/1000 kcal)	-0.44***	0.25**	0.51***
Vitamin C (mg/1000 kcal)	-0.23**	0.36***	0.61***
Folate-Equivalents (µg/1000 kcal)	-0.32***	0.23**	0.43***
Vitamin B_1_ (µg/1000 kcal)	-0.40***	0.53***	0.34***
Vitamin B_2_ (µg/1000 kcal)	0.45***	-0.17*	0.15
Vitamin B_12_ (µg/1000 kcal)	0.73***	-0.42***	-0.08
Calcium (mg/1000 kcal)	0.49***	-0.09	0.04
Magnesium (mg/1000 kcal)	-0.45***	0.60***	0.42***
Iron (mg/1000 kcal)	-0.50***	0.46***	0.37***
Zinc (mg/1000 kcal)	0.09	0.38***	0.02

Correlations significant at * *P* < 0.05, **P < 0.01 and ***P < 0.001 (H0: *r* = 0); strong correlations (|r| ≥ 0.50) are shown in bold MUFA, mono-unsaturated fatty acids, PUFA, polyunsaturated fatty acids; SFA, saturated fatty acids

**Table 3:** Spearman correlation between dietary pattern scores and energy and nutrient density in vegan participants of the German VeChi Youth Study (*n* = 110)

	Vegetables and legumes	Refined carbohydrates	Meat alternatives and juices	Fruits and convenience food
Energy (kcal/100 g)	-0.64***	-0.06	0.03	-0.53***
Fat (%E)	-0.34***	0.13	0.09	-0.52***
SFA (%E)	-0.21*	0.35***	0.19*	-0.53***
MUFA (%E)	-0.26**	0.03	-0.15	-0.43***
PUFA (%E)	-0.37***	-0.17	0.12	-0.22*
Linoleic acid (%E)	-0.39***	-0.16	0.21*	-0.20*
α-Linolenic acid (%E)	-0.10	-0.06	-0.03	-0.20*
Protein (%E)	0.04	-0.48***	0.00	0.41***
Carbohydrates (%E)	0.32***	0.04	-0.09	0.36***
Added sugars (%E)	-0.14	0.29**	0.01	-0.35***
Dietary fiber (g/1000 kcal)	0.31***	-0.60***	-0.36***	0.67***
Retinol-Equivalents (µg/1000 kcal)	0.43***	0.02	-0.04	0.14
Tocopherol-Equivalents (µg/1000 kcal)	-0.08	-0.08	0.18	-0.09
Vitamin C (mg/1000 kcal)	0.51***	-0.24*	-0.12	0.50***
Folate-Equivalents (µg/1000 kcal)	0.26**	-0.42***	-0.15	0.48***
Vitamin B_1_ (µg/1000 kcal)	0.21*	-0.68***	-0.34***	0.40***
Vitamin B_2_ (µg/1000 kcal)	0.23*	-0.34***	0.01	0.45***
Vitamin B_12_ (µg/1000 kcal)	-0.04	0.19*	0.23**	-0.13
Calcium (mg/1000 kcal)	0.16	-0.34***	0.01	0.43***
Magnesium (mg/1000 kcal)	0.28**	-0.64***	-0.45***	0.55***
Iron (mg/1000 kcal)	0.22*	-0.66***	-0.31***	0.36***
Zinc (mg/1000 kcal)	0.15	-0.69***	-0.46***	0.45***

Correlations significant at * *P* < 0.05, **P < 0.01 and ***P < 0.001 (H0: *r* = 0); strong correlations (|r| ≥ 0.50) are shown in bold MUFA, mono-unsaturated fatty acids, PUFA, polyunsaturated fatty acids; SFA, saturated fatty acids

### Vegetarian diet group

Table 2 presents the correlations between dietary pattern scores and energy and nutrient density in the vegetarian diet group. Both the “Vegetables and fruits” and the “Meat alternatives and potatoes” patterns showed moderate negative correlations with energy density (*r* = -0.32 and *r* = − 0,37). No significant correlation was found between the “Animal foods” pattern and energy density. Higher scores on the vegetarian “Animal foods” pattern were strongly correlated with fat quality (higher saturated fatty acids, *r* = 0.60; lower polyunsaturated fatty acids, *r* = -0.54 and linoleic acid *r* = -0.57). Strong negative correlations were also seen for nutrient density of dietary fiber (*r* = -0.65) and iron (*r* = -0.50) and a strong positive correlation for vitamin B_12_ density (*r* = 0.73). The “Vegetables and fruits” pattern showed strong positive correlations with dietary fiber (*r* = 0.69), vitamin B_1_ (*r* = 0.53) and magnesium (*r* = 0.60). Higher scores on the ‘Meat alternatives and potatoes’ pattern were associated with greater tocopherol-equivalents (r = 0.51) and vitamin C density (r = 0.61) in the diet of the vegetarian children and adolescents.

### Vegan diet group

Table 3 shows the correlations between dietary pattern scores and energy and nutrient density in the vegetarian diet group. Strong negative correlations with energy density were seen for the dietary patterns “Vegetables and legumes” (r= -0.64) and “Fruits and convenience food” (r= -0.53). The “Refined carbohydrates” and “Meat alternatives and juices” patterns showed no significant correlations with energy density. Higher scores on the vegan “Vegetables and legumes” pattern were strongly correlated with vitamin C density (r = 0.51). The pattern „Refined carbohydrates” was strongly negatively correlated with dietary fiber (*r*= -0.60), vitamin B_1_ (*r*= -0.68), magnesium (*r*= -0.64), iron (*r*= -0.66) and zinc (*r*= -0.69). The “fruits and convenience food” pattern showed strong positive correlations with dietary fiber (*r* = 0.67) and magnesium (*r* = 0.55) and strong negative correlations with total fat (*r*= -0.52) and saturated fatty acids (*r*= -0.53). No strong correlations were found between the “Meat alternatives and juices” pattern and any of the nutrients, but moderate negative correlations were found with dietary fiber (*r*= -0.36), vitamin B_1_ (*r*= -0.34), magnesium (*r*= -0.45), iron (*r*= -0.31) and zinc (*r*= -0.46).

**Table 4:** Spearman correlation between dietary pattern scores and energy and nutrient density in omnivore participants of the German VeChi Youth Study (*n* = 135)

	Flexitarian	Vegetables and fruits	Dairy products	Meat and convenience food	Refined grains and juices
Energy (kcal/100 g)	-0.17	-0.63***	0.31***	-0.23**	0.07
Fat (%E)	-0.02	-0.22*	0.24**	0.01	-0.26**
SFA (%E)	-0.13	-0.26**	0.45***	-0.06	-0.26**
MUFA (%E)	-0.01	-0.21*	0.11	0.11	-0.15
PUFA (%E)	0.28***	0.08	-0.32***	-0.05	-0.12
Linoleic acid (%E)	0.29***	0.01	-0.30***	-0.02	-0.09
α-Linolenic acid (%E)	0.26**	0.22*	-0.28***	-0.18*	-0.23**
Protein (%E)	0.06	0.38***	0.05	0.53***	0.06
Carbohydrates (%E)	0.02	0.08	-0.25**	-0.24**	0.22*
Added sugars (%E)	-0.44***	-0.57***	0.01	0.01	-0.13
Dietary fiber (g/1000 kcal)	0.62***	0.67***	-0.33***	-0.38***	-0.24**
Retinol-Equivalents (µg/1000 kcal)	0.29***	0.30***	-0.18*	-0.13	-0.32***
Tocopherol-Equivalents (µg/1000 kcal)	0.35***	0.22*	-0.34***	-0.07	-0.11
Vitamin C (mg/1000 kcal)	0.16	0.49***	-0.26**	-0.22**	-0.09
Folate-Equivalents (µg/1000 kcal)	0.35***	0.52***	-0.28***	-0.06	-0.11
Vitamin B_1_ (µg/1000 kcal)	0.33***	0.27**	-0.03	0.12	0.13
Vitamin B_2_ (µg/1000 kcal)	-0.06	0.20*	0.19*	0.21*	-0.10
Vitamin B_12_ (µg/1000 kcal)	-0.32***	0.03	0.23**	0.50***	0.07
Calcium (mg/1000 kcal)	0.11	0.26**	0.10	-0.06	-0.24**
Magnesium (mg/1000 kcal)	0.63***	0.53***	-0.20*	-0.20*	-0.26**
Iron (mg/1000 kcal)	0.57***	0.40***	-0.24**	-0.09	-0.24**
Zinc (mg/1000 kcal)	0.28***	0.49***	0.06	0.30***	-0.07

Correlations significant at * *P* < 0.05, **P < 0.01 and ***P < 0.001 (H0: *r* = 0); strong correlations (|r| ≥ 0.50) are shown in bold MUFA, mono-unsaturated fatty acids, PUFA, polyunsaturated fatty acids; SFA, saturated fatty acids

### Omnivore diet group

Table 4 presents the correlations between dietary pattern scores and energy and nutrient density in the omnivore diet group. The “Vegetables and fruits” pattern showed strong negative correlations with energy density (*r*= -0.63), whereas the “Dairy products” showed moderate positive correlations with energy density (*r* = 0.31). Higher scores on the “Flexitarian” dietary pattern were strongly correlated with dietary fiber (*r* = 0.62), magnesium (*r* = 0.63) and iron density (*r* = 0.57). The “Vegetables and fruits” pattern showed strong positive correlations with dietary fiber (*r* = 0.67), folate-equivalents (*r* = 0.52) and magnesium density (*r* = 0.53) as well as strong negative correlations with added sugars (*r*= -0.57). The “Meat and convenience food” correlated strongly with protein density (*r* = 0.53). No strong correlations were found between the “Refined grains and juices” pattern and any of the nutrients.

## Discussion

In the cross-sectional VeChi Youth Study, different dietary patterns were identified not only among omnivore children and adolescents, but also within the vegetarian and vegan diet groups. These dietary patterns were differentially associated with nutrient intake. Regardless of diet group, we observed both, more health-conscious patterns as well as less favorable patterns. The dietary patterns “Vegetables and fruits” (vegetarian and omnivore), “Meat alternatives and potatoes” (vegetarian), “Vegetables and legumes” (vegan), “Fruits and convenience food” (vegan) and “Flexitarian” (omnivore) correlated with higher micronutrient density and therefore seem more health-conscious. In contrast, the patterns “Animal foods” (vegetarian), “Refined carbohydrates” (vegan), “Meat alternatives and juices” (vegan), “Dairy products” (omnivore), “Meat and convenience food” (omnivore) and “Refined grains and juices” (omnivore) were associated with a less favorable micronutrient profile and/or lower intakes of potentially critical nutrients.

Many studies using *a posteriori* dietary pattern analysis found various dietary patterns among omnivore children and adolescents in Western countries [22–37]. Similar to the present study, these studies were able to identify at least one beneficial pattern considered as healthy, which was characterized by high consumption of unprocessed or minimally processed plant-based foods, such as vegetables, fruits, legumes, whole grains, or nuts [22–25, 27–34]. In addition, consistent with the VeChi Youth Study, less favorable dietary patterns were identified, that focus on energy-dense and highly processed foods, such as fast food, convenience foods, snacks, sweets or sugar sweetened beverages [23–27, 29, 30, 32, 34, 35].

The two predominant dietary patterns in the omnivore diet group, “Flexitarian” and “Vegetables and fruits”, were both characterized by a high intake of plant-based foods, including meat- and dairy alternatives. This suggests that plant-based dietary patterns are also common among children and adolescents classified as “omnivore”. This would be consistent with the results of several studies that found increasing popularity of flexitarian diets in European countries [2, 56, 57]. Since current research [58] as well as our results show substantial differences between flexitarians and traditional omnivores in terms of food selection, nutrient intake and nutrition status [58], a differentiation between flexitarian and traditional omnivore diets seems to be useful.

To date, *a posteriori* dietary patterns within vegetarian or vegan diets have only been studied among adult vegans [38, 39], but the dietary patterns in these studies show certain similarities with the vegan dietary patterns found in the VeChi Youth study. In a British study with 129 adult vegans (87% female, age 18–64 years) Gallagher et al. were able to identify four different vegan dietary patterns using PCA (based on 20 food groups), the patterns „Convenience“, „Health conscious“, „Unhealthy“ and „Traditional vegan“ [39]. In a recent Austrian study with 516 vegans (mean age (SD) 28.0 (7.7), 85% female), Haider et al. identified two dietary patterns using PCA (based on 18 food groups), a “Convenience” and a “Health-conscious” pattern [38]. The “Vegetables and legumes” pattern in the VeChi Youth study shows clear parallels with the “Traditional vegan” pattern found in British vegans [39] and the “Health Conscious” pattern in Austrian vegans [38]. All three dietary patterns focus on unprocessed plant foods, especially vegetables, fruits, and potatoes. In accordance with the VeChi Youth Study, Gallagher et al. and Haider et al. also extracted more unfavorable dietary patterns [38, 39]. In particular, the “Refined carbohydrates” pattern in the VeChi Youth study shows parallels with the unhealthier “Convenience” patterns found by Gallagher et al. [39] and Haider et al. [38], as all of these patterns are characterized by higher intake of vegan convenience foods, snacks, sweets and ultra-processed food items. These similarities between our extracted dietary patterns and the vegan dietary patterns in other studies confirm the results of our pattern analysis and show that comparable vegan dietary patterns can be found in vegan children/adolescents and adults.

Unfavorable vegan dietary patterns are associated with a lower intake of legumes, whole grains, nuts, and seeds. However, sufficient intake of these food groups is of particular importance to ensure adequate supply of potentially critical nutrients (such as protein, iron, zinc, calcium, and Vitamin B_2_) in a vegan diet [18, 19]. In the VeChi Youth Study, the vegan subgroup was found to have the highest overall consumption of these food groups [42]. However, our analysis showed that legumes did not occur equally in all vegan dietary patterns. Only the “Vegetables and legumes” pattern was associated with higher legume consumption, whereas the “Meat alternatives and potatoes” pattern was characterized by lower consumption. Thus, with increasing consumption of meat alternatives, legumes appear to be neglected as a protein source. Higher consumption of whole grains, nuts, and seeds did not distinguish any of the identified patterns, but the “Refined carbohydrates” and “Meat alternatives and juices” patterns were associated with lower consumption of the two food groups. More unfavorable nutrient correlations were also found for these two dietary patterns, once again highlighting the importance of these food groups in providing critical nutrients.

Many of the associations between dietary patterns and energy and nutrient intake in the VeChi Youth study can also be found in other studies with omnivore children and adolescents [22, 25, 30, 37], supporting the findings of our analysis. Across these studies *a posteriori* dietary patterns considered healthier were associated with a more beneficial nutrient profile [22, 25, 30, 37]. As the vegetable and fruit-rich patterns in the VeChi Youth Study, the “Health conscious” or “Traditional/health-conscious” dietary patterns identified in 3-9-year-old children [30] and 13-year-old adolescents [37] in the Avon Longitudinal Study of Parents and Children (ALSPAC), were correlated with higher energy-adjusted intakes of dietary fiber and most micronutrients [30, 37]. Ambrosini et al. [22] also found these correlations in 13-year-old adolescents in the Australian Raine study. In addition, there was an inverse association between the “Healthy” pattern and energy-adjusted saturated fat intake [22], as was also shown in all three dietary groups in the VeChi Youth study. The same correlations between the above-mentioned nutrients and the identified “Healthy” patterns were also shown by Richter et al. [25] in 12- to 17-year-old girls and boys in the EsKiMo.

Similarly to other studies, the dietary patterns in the VeChi Youth study considered as unhealthier were associated with a more unfavorable nutrient profile. The vegan pattern “Refined carbohydrates” in the VeChi Youth Study showed particularly high agreement in nutrient correlations with the unfavorable dietary patterns “Snacks/sugared drinks” in the ALSPAC [37] and “Western” in the Raine study [22]. The three patterns were similar not only in the high factor loadings for sweets, snack foods, and sugar-sweetened beverages, but also correlated with higher total sugar intake and lower intakes of protein, dietary fiber, and most micronutrients (especially magnesium, iron, and zinc) [22, 37].

Patterns with individual food groups, which are classified as unfavorable due to their high degree of processing, however, cannot be clearly classified as unhealthy. In the present work, e.g. the dietary pattern “Fruits and convenience food” was the only vegan pattern to show significant positive correlations with the intake of the critical nutrients protein, zinc and calcium. This may due to the fact, that several plant-based dairy alternatives are fortified with minerals, e.g. calcium (27). In addition, negative correlations with the intake of energy, added sugars and saturated fatty acids were observed. This underlines that vegan convenience foods or more highly processed plant-based alternative products are not to be classified as unfavorable per se, but, depending on their ingredients and nutrient enrichment, can contribute to an adequate intake of critical nutrients. With increasing consumption of meat alternatives, legumes appear to be neglected as a protein source. Thus, our results can contribute to the debate about the composition of meat- and dairy alternatives and whether they should be equated to other highly processed foods [59–62].

The strengths of the VeChi Youth Study include the detailed dietary survey using 3-day weighed dietary records, in which fortified foods as well as plant-based convenience foods and alternative products were taken into account. Another strength is the large study population of vegetarian and vegan children and adolescents, including an omnivore control group, with equal age distribution and matching sociodemographic characteristics of the three diet groups.

However, the analysis also has some limitations. Due to the above-average socioeconomic status of the study participants and the regional focus in North Rhine-Westphalia, the VeChi Youth Study is not representative and the results can therefore only be generalized to a limited extent. However, the high socioeconomic status as well as the high proportion of urban residents in the study population correspond to the known sociodemographic characteristics of vegetarians and vegans [3]. In contrast, the rather high socioeconomic status in the omnivore group is probably due to selection bias [42]. Thus, in a representative study, potentially larger differences between dietary patterns and associations with health-related outcomes would be detectable.

The sample size for the pattern analysis was relatively small, as the power calculation was carried out for a different outcome, which may also affect the representativeness of our results. Overall measure of sampling adequacy according to KMO indicated insufficient suitability of the data for PCA, however the observed patterns seem plausible for vegan, vegetarian and omnivore diets. In addition, in order to obtain reliable results in the PCA, it is recommended that the number of study participants should be at least five times as large as the number of analyzed variables (food groups) [50, 63], which we were able to fulfil in our analyses. Furthermore, Bartlett test for sphericity showed a significant result in all three diet groups (*P* < 0.001), which indicates the adequacy of the data for PCA. Due to the small sample size, it was not possible to perform stratified analyses in addition to stratification of the diet group and thus to determine age- or sex-specific differences in dietary patterns. Therefore, we could not rule out the possibility that different age- and sex-distributions in the diet groups may have influenced our results. However, prospective studies with omnivore populations have found consistent dietary patterns from childhood to adolescence [24, 30, 64] and very similar patterns in younger girls and boys [27, 28, 32]. In adolescents, on the other hand, different dietary patterns have been found according to sex [25, 26, 65]. The extent to which vegetarian or vegan children and adolescents show age- or sex-specific differences in their dietary patterns is not known and should therefore be investigated in future studies.

With regard to the dietary assessment, it should be mentioned that the record duration of 3 days might not be sufficient for recording the participants’ habitual diet. However, more records days are discussed to may cause a decline in the quality of information [66]. As the participants were free to choose the days of the dietary records, it cannot be ruled out that the records were also completed on uncommon, unrepresentative days, such as holidays. Moreover, the dietary records were completed on three consecutive days, but at different times of the year. It is therefore possible that seasonal differences in the participants’ diet al.so had an impact on the results.

A general limitation of PCA is subjectivity, especially when aggregating food groups, determining the number of patterns to be derived, and labelling the components [67]. The population specificity also makes the identified dietary patterns difficult to reproduce. Therefore, to increase comparability with other studies, a methodological approach analogous to that used in other studies was adopted in extracting the dietary patterns [25, 26]. Furthermore, dietary patterns extracted by PCA are only able to explain a small proportion of variance in diets [68]. In the present evaluation, the individual dietary patterns extracted based on 17, 21, and 22 food groups could each explain only 7.4–13.0% of the variance in food group intake. However, these proportions of variance are consistent with the results of other studies in children [27, 35] or adolescents [34, 36].

Concerning the investigated nutrient correlations, it should be mentioned that not all potentially critical nutrients in vegetarian or vegan diets could be considered in the analysis and thus, for example, information on the intake of iodine, selenium and long-chain omega-3 fatty acids was missing. For iodine, this is due to the fact that table salt as the main source of iodine was not quantitatively assessed. Data on selenium are not available in German food composition tables and databases from other countries are not useful as selenium content in foods depends on geography. Sources of long-chain omega-3 fatty acids in vegan diets are fortified foods and supplements, but the latter are not included here. Supplementary studies with investigations on these nutrients are therefore desirable. Moreover, the sole investigation of nutrient intake does not allow any conclusion to be drawn on the actual nutrient status of the study participants. The inclusion of biomarkers for nutrient status and longitudinal studies could provide further insights into the relationship between dietary patterns and nutritional status.

## Conclusion

The present study shows different dietary patterns that vary in their contribution to nutrient intake, not only in omnivores but also in vegetarian and vegan children and adolescents. Regardless of diet type, there appear to be both more nutritionally favorable plant-based dietary patterns associated with higher micronutrient density and more nutritionally unfavorable dietary patterns with a greater focus on energy-dense and/or more highly processed foods. In the vegetarian and vegan diet group, the nutrient correlations suggest that certain dietary patterns characterized by a lower intake of legumes, whole grains and/or nuts and seeds are associated with a lower intake of critical nutrients. This emphasizes the importance of these food groups for adequate nutrient intake in vegetarian and vegan diets.

There are many similarities between the vegetarian, vegan and omnivore diet groups in terms of the composition and nutritional properties of the patterns identified. This indicates that certain dietary patterns occur independently of the form of diet. A general nutritional evaluation of the diet based on the conventional classification into “vegetarian”, “vegan” or “omnivore” is therefore only of limited use. Rather, a differentiation between nutritionally more favorable and less favorable dietary patterns within the form of diet seems appropriate. Prospective studies are desirable to verify the consistency of the identified dietary patterns and to assess their long-term preventive relevance.

## Electronic supplementary material

Below is the link to the electronic supplementary material.

Supplementary Material 1

## Data Availability

Data of the VeChi Youth Study are available on request to alexy@uni-bonn.de.

## Abbreviations

ALSPAC: Avon longitudinal study of parents and children
EsKiMo: German representative second eating study as a KiGGS module
KMO: Kaiser-Meyer-Olkin
PCA: principal component analysis
VeChi Youth Study: Vegetarian and vegan children and Youth Study

## References

[1] Robert Koch-Institut (2016) Verbreitung der vegetarischen Ernährungsweise in Deutschland. RKI-Bib1 (Robert Koch-Institut)

[2] Bundesministeriums für Ernährung und Landwirtschaft (2022) Deutschland, wie es isst: Der BMEL-Ernährungsreport 2022. https://www.bmel.de/SharedDocs/Downloads/DE/Broschueren/ernaehrungsreport-2022.pdf?__blob=publicationFile&v=6. Accessed 11 Nov 2022

[3] Patelaki E, Lage Barbosa C, Haftenberger M et al (2019) Prevalence of vegetarian diet among children and adolescents in Germany. Results from EsKiMo II. Ernahrungs Umschau 85–91. 10.4455/eu.2019.018

[4] Hemler EC, Hu FB (2019) Plant-based diets for Cardiovascular Disease Prevention: all Plant Foods are not created equal. Curr Atheroscler Rep 21:18. 10.1007/s11883-019-0779-5 30895476 10.1007/s11883-019-0779-5

[5] Appleby PN, Key TJ (2016) The long-term health of vegetarians and vegans. Proc Nutr Soc 75:287–293. 10.1017/S0029665115004334 26707634 10.1017/S0029665115004334

[6] Benatar JR, Stewart RAH (2018) Cardiometabolic risk factors in vegans; a meta-analysis of observational studies. PLoS ONE 13:e0209086. 10.1371/journal.pone.0209086 30571724 10.1371/journal.pone.0209086 PMC6301673

[7] Dinu M, Abbate R, Gensini GF et al (2017) Vegetarian, vegan diets and multiple health outcomes: a systematic review with meta-analysis of observational studies. Crit Rev Food Sci Nutr 57:3640–3649. 10.1080/10408398.2016.1138447 26853923 10.1080/10408398.2016.1138447

[8] Wang F, Zheng J, Yang B et al (2015) Effects of vegetarian diets on blood lipids: a systematic review and Meta-analysis of Randomized controlled trials. J Am Heart Assoc 4:e002408. 10.1161/JAHA.115.002408 26508743 10.1161/JAHA.115.002408 PMC4845138

[9] Yokoyama Y, Levin SM, Barnard ND (2017) Association between plant-based diets and plasma lipids: a systematic review and meta-analysis. Nutr Rev 75:683–698. 10.1093/nutrit/nux030 28938794 10.1093/nutrit/nux030 PMC5914369

[10] Ocagli H, Berti G, Rango D et al (2023) Association of Vegetarian and Vegan Diets with Cardiovascular Health: an Umbrella Review of Meta-Analysis of Observational studies and Randomized trials. Nutrients 15. 10.3390/nu15194103 10.3390/nu15194103 PMC10574056 37836394

[11] Koller A, Rohrmann S, Wakolbinger M et al (2023) Health aspects of vegan diets among children and adolescents: a systematic review and meta-analyses. Crit Rev Food Sci Nutr 1–12. 10.1080/10408398.2023.2263574 10.1080/10408398.2023.2263574 37811643

[12] Neufingerl N, Eilander A (2021) Nutrient intake and status in adults consuming plant-based diets compared to Meat-Eaters: a systematic review. Nutrients 14. 10.3390/nu14010029 10.3390/nu14010029 PMC8746448 35010904

[13] Rudloff S, Bührer C, Jochum F et al (2019) Vegetarian diets in childhood and adolescence: position paper of the nutrition committee, German Society for Paediatric and Adolescent Medicine (DGKJ). Mol Cell Pediatr 6:4. 10.1186/s40348-019-0091-z 31722049 10.1186/s40348-019-0091-z PMC6854160

[14] Richter M, Boeing H, Grünewald-Funk D, Heseker H, Kroke A, Leschik-Bonnet E (2016) Vegane Ernährung Position Der Deutschen Gesellschaft für Ernährung e. V. (DGE). Ernährungs Umschau 63:M220–M230

[15] Rivera JA, Hotz C, González-Cossío T et al (2003) The effect of micronutrient deficiencies on child growth: a review of results from community-based supplementation trials. J Nutr 133. 10.1093/jn/133.11.4010S. 4010S-4020S 10.1093/jn/133.11.4010S 14672304

[16] Bryan J, Osendarp S, Hughes D et al (2004) Nutrients for cognitive development in school-aged children. Nutr Rev 62:295–306. 10.1111/j.1753-4887.2004.tb00055.x 15478684 10.1111/j.1753-4887.2004.tb00055.x

[17] Georgieff MK (2007) Nutrition and the developing brain: nutrient priorities and measurement. Am J Clin Nutr 85. 10.1093/ajcn/85.2.614S. 614S-620S 10.1093/ajcn/85.2.614S 17284765

[18] Alexy U, Keller M, Straub S (2019) Vegetarische oder vegane Ernährung in Der Kindheit – was ist zu Beachten? Kinder- und Jugendarzt 50:240–245

[19] Marczykowski F, Breidenassel C (2017) Vegan diet: reaching the reference values for nutrient intake of critical nutrients: assortment and necessity of fortified foods. Ernährungs Umschau 64. 10.4455/eu.2017.002

[20] Hu FB (2002) Dietary pattern analysis: a new direction in nutritional epidemiology. Curr Opin Lipidol 13:3–9. 10.1097/00041433-200202000-00002 11790957 10.1097/00041433-200202000-00002

[21] Newby PK, Tucker KL (2004) Empirically derived eating patterns using factor or cluster analysis: a review. Nutr Rev 62:177–203. 10.1301/nr.2004.may.177-203 15212319 10.1301/nr.2004.may.177-203

[22] Ambrosini GL, O’Sullivan TA, de Klerk NH et al (2011) Relative validity of adolescent dietary patterns: a comparison of a FFQ and 3 d food record. Br J Nutr 105:625–633. 10.1017/S0007114510004137 21269548 10.1017/S0007114510004137 PMC3308192

[23] Flynn AC, Thompson JMD, Dalrymple KV et al (2020) Childhood dietary patterns and body composition at age 6 years: the children of SCOPE study. Br J Nutr 1–21. 10.1017/S0007114520000628 10.1017/S0007114520000628 PMC7116586 32098635

[24] Northstone K, Smith ADAC, Newby PK et al (2013) Longitudinal comparisons of dietary patterns derived by cluster analysis in 7- to 13-year-old children. Br J Nutr 109:2050–2058. 10.1017/S0007114512004072 23068994 10.1017/S0007114512004072

[25] Richter A, Heidemann C, Schulze MB et al (2012) Dietary patterns of adolescents in Germany–associations with nutrient intake and other health related lifestyle characteristics. BMC Pediatr 12:35. 10.1186/1471-2431-12-35 22439777 10.1186/1471-2431-12-35 PMC3386018

[26] Richter A, Rabenberg M, Truthmann J et al (2017) Associations between dietary patterns and biomarkers of nutrient status and cardiovascular risk factors among adolescents in Germany: results of the German health interview and examination survey for children and adolescents in Germany (KiGGS). BMC Nutr 3. 10.1186/s40795-016-0123-1

[27] Wolters M, Joslowski G, Plachta-Danielzik S et al (2018) Dietary patterns in Primary School are of prospective relevance for the development of body composition in two German Pediatric populations. 10.3390/nu10101442. Nutrients 10 10.3390/nu10101442 PMC6213904 30301151

[28] Diethelm K, Günther ALB, Schulze MB et al (2014) Prospective relevance of dietary patterns at the beginning and during the course of primary school to the development of body composition. Br J Nutr 111:1488–1498. 10.1017/S0007114513004017 24382029 10.1017/S0007114513004017

[29] Araújo J, Teixeira J, Gaio AR et al (2015) Dietary patterns among 13-y-old Portuguese adolescents. Nutrition 31:148–154. 10.1016/j.nut.2014.06.007 25466659 10.1016/j.nut.2014.06.007

[30] Cribb V, Emmett P, Northstone K (2013) Dietary patterns throughout childhood and associations with nutrient intakes. Public Health Nutr 16:1801–1809. 10.1017/S1368980012004132 22974523 10.1017/S1368980012004132 PMC10271259

[31] Smith ADAC, Emmett PM, Newby PK et al (2013) Dietary patterns obtained through principal components analysis: the effect of input variable quantification. Br J Nutr 109:1881–1891. 10.1017/S0007114512003868 22950853 10.1017/S0007114512003868

[32] Kanellopoulou A, Kosti RI, Notara V et al (2021) Dietary patterns, weight perception and obesity status, among 10-12-year-old children; an epidemiological study in Greece. Child (Basel) 8. 10.3390/children8080626 10.3390/children8080626 PMC8393401 34438517

[33] Barchitta M, Maugeri A, Agrifoglio O et al (2019) Dietary patterns and school performance: evidence from a sample of adolescents in Sicily, Italy. Ann Ig 31:72–80. 10.7416/ai.2019.2279 30994166 10.7416/ai.2019.2279

[34] Wadolowska L, Kowalkowska J, Lonnie M et al (2016) Associations between physical activity patterns and dietary patterns in a representative sample of Polish girls aged 13–21 years: a cross-sectional study (GEBaHealth Project). BMC Public Health 16:698. 10.1186/s12889-016-3367-4 27485607 10.1186/s12889-016-3367-4 PMC4971681

[35] Plaza-Díaz J, Molina-Montes E, Soto-Méndez MJ et al (2020) Clustering of Dietary Patterns and Lifestyles Among Spanish Children in the EsNuPI Study †. Nutrients 12. 10.3390/nu12092536 10.3390/nu12092536 PMC7551863 32825604

[36] Kafyra M, Kalafati IP, Kumar S et al (2021) Dietary patterns, blood pressure and the Glycemic and Lipidemic Profile of two teenage, European populations. Nutrients 13 10.3390/nu13010198 10.3390/nu13010198 PMC7826952 33435217

[37] Northstone K, Smith ADAC, Cribb VL et al (2014) Dietary patterns in UK adolescents obtained from a dual-source FFQ and their associations with socio-economic position, nutrient intake and modes of eating. Public Health Nutr 17:1476–1485. 10.1017/S1368980013001547 23782861 10.1017/S1368980013001547 PMC10282453

[38] Haider S, Sima A, Kühn T et al (2023) The Association between vegan dietary patterns and physical Activity-A cross-sectional online survey. Nutrients 15 10.3390/nu15081847 10.3390/nu15081847 PMC10145789 37111067

[39] Gallagher CT, Hanley P, Lane KE (2021) Pattern analysis of vegan eating reveals healthy and unhealthy patterns within the vegan diet. Public Health Nutr 1–11. 10.1017/S136898002100197X 10.1017/S136898002100197X PMC9991567 33971998

[40] Alexy U, Fischer M, Weder S, Längler A, Michalsen A (2020) Vegetarische und vegane Ernährung Bei Kindern Und Jugendlichen in Deutschland – VeChi-Youth-Studie. DGE-Ernährungsbericht, Bonn, pp 289–354, p 14. Deutsche Gesellschaft für Ernährung

[41] Alexy U, Fischer M, Weder S et al (2021) Nutrient intake and status of German children and adolescents consuming vegetarian, vegan or Omnivore diets: results of the VeChi Youth Study. Nutrients 13. 10.3390/nu13051707 10.3390/nu13051707 PMC8157583 34069944

[42] Alexy U, Fischer M, Weder S et al (2021) Food group intake of children and adolescents (6–18 years) on a vegetarian, vegan, or omnivore diet: results of the VeChi Youth Study. Br J Nutr 1–26. 10.1017/S0007114521003603 10.1017/S0007114521003603 34511141

[43] Koebnick C, Garcia AL, Dagnelie PC et al (2005) Long-term consumption of a raw food diet is associated with favorable serum LDL cholesterol and triglycerides but also with elevated plasma homocysteine and low serum HDL cholesterol in humans. J Nutr 135:2372–2378. 10.1093/jn/135.10.2372 16177198 10.1093/jn/135.10.2372

[44] Winkler J, Stolzenberg H (2009) Adjustierung Des Sozialen-Schicht-Index für die Anwendung Im Kinder- Und Jugendgesundheitssurvey (KiGGS) 2003/2006. Wismarer Diskussionspapiere, 2009, H. 7. Hochsch. Fakultät für Wirtschaftswiss, HWS-Hochsch.-Wismar-Service, Wismar

[45] Booth ML, Okely AD, Chey TN et al (2002) The reliability and validity of the adolescent physical activity recall Questionnaire. Med Sci Sports Exerc 34:1986–1995. 10.1097/00005768-200212000-00019 12471306 10.1097/00005768-200212000-00019

[46] Ainsworth BE, Haskell WL, Herrmann SD et al (2011) 2011 Compendium of Physical activities: a second update of codes and MET values. Med Sci Sports Exerc 43:1575–1581. 10.1249/MSS.0b013e31821ece12 21681120 10.1249/MSS.0b013e31821ece12

[47] Butte NF, Watson KB, Ridley K et al (2018) A Youth Compendium of Physical activities: Activity codes and metabolic intensities. Med Sci Sports Exerc 50:246–256. 10.1249/MSS.0000000000001430 28938248 10.1249/MSS.0000000000001430 PMC5768467

[48] Kromeyer-Hauschild K, Moss A, Wabitsch M (2015) Referenzwerte für den body-Mass-Index für Kinder, Jugendliche Und Erwachsene in Deutschland. Adipositas - Ursachen. Folgeerkrankungen Ther 09:123–127. 10.1055/s-0037-1618928

[49] Sichert-Hellert W, Kersting M, Chahda C et al (2007) German food composition database for dietary evaluations in children and adolescents. J Food Compos Anal 20:63–70. 10.1016/j.jfca.2006.05.004

[50] O’Rourke N, Hatcher L (2014) A Step-by-Step Approach to Using SAS for Factor Analysis and Structural Equation Modeling, Second Edition, Cary, North Carolina

[51] Hatcher L (2007) A Step-by-Step Approach to Using the SAS System for Factor Analysis and Structural Equation Modeling. 9 edition, Cary, North Carolina

[52] Schulze MB, Hoffmann K, Kroke A et al (2001) Dietary patterns and their association with food and nutrient intake in the European prospective investigation into Cancer and Nutrition (EPIC)-Potsdam study. Br J Nutr 85:363–373. 10.1079/bjn2000254 11299082 10.1079/bjn2000254

[53] Willett WC, Howe GR, Kushi LH (1997) Adjustment for total energy intake in epidemiologic studies. Am J Clin Nutr 65. 10.1093/ajcn/65.4.1220S. 1220S-1228S; discussion 1229S-1231S 10.1093/ajcn/65.4.1220S 9094926

[54] Hernes S, Cabo RN, Mansoor MA et al (2012) Eating patterns are associated with biomarkers in a selected population of university students and employees. J Nutr Sci 1:e8. 10.1017/jns.2012.8 25191555 10.1017/jns.2012.8 PMC4153286

[55] Cohen J (2013) Statistical Power Analysis for the behavioral sciences, 2nd edn. Taylor and Francis, Hoboken

[56] Veganz Group AG (2021) veganz Ernährungsreport: Zahlen 2021. https://veganz.de/wp-content/uploads/2021/11/20211101-veganz-ernaehrungsreport-2021.pdf. Accessed 11 Nov 2022

[57] Deliens T, Mullie P, Clarys P (2022) Plant-based dietary patterns in flemish adults: a 10-year trend analysis. Eur J Nutr 61:561–565. 10.1007/s00394-021-02630-z 34213604 10.1007/s00394-021-02630-z

[58] Dawczynski C, Weidauer T, Richert C et al (2022) Nutrient intake and Nutrition Status in vegetarians and vegans in comparison to omnivores - the nutritional evaluation (NuEva) study. Front Nutr 9:819106. 10.3389/fnut.2022.819106 35651513 10.3389/fnut.2022.819106 PMC9149309

[59] Craig WJ, Messina V, Rowland I et al (2023) Plant-based dairy Alternatives Contribute to a healthy and sustainable Diet. 10.3390/nu15153393. Nutrients 15 10.3390/nu15153393 PMC10421454 37571331

[60] Gehring J, Touvier M, Baudry J et al (2021) Consumption of Ultra-processed Foods by Pesco-Vegetarians, vegetarians, and vegans: associations with Duration and Age at Diet initiation. J Nutr 151:120–131. 10.1093/jn/nxaa196 32692345 10.1093/jn/nxaa196

[61] Sridhar K, Bouhallab S, Croguennec T et al (2023) Recent trends in design of healthier plant-based alternatives: nutritional profile, gastrointestinal digestion, and consumer perception. Crit Rev Food Sci Nutr 63:10483–10498. 10.1080/10408398.2022.2081666 35647889 10.1080/10408398.2022.2081666

[62] Flint M, Bowles S, Lynn A et al (2023) Novel plant-based meat alternatives: future opportunities and health considerations. Proc Nutr Soc 82:370–385. 10.1017/S0029665123000034 36603854 10.1017/S0029665123000034

[63] Streiner DL (1994) Figuring out factors: the use and misuse of factor analysis. Can J Psychiatry 39:135–140. 10.1177/070674379403900303 8033017 10.1177/070674379403900303

[64] Mikkilä V, Räsänen L, Raitakari OT et al (2005) Consistent dietary patterns identified from childhood to adulthood: the cardiovascular risk in Young finns Study. Br J Nutr 93:923–931. 10.1079/bjn20051418 16022763 10.1079/bjn20051418

[65] González-Gil EM, Martínez-Olivan B, Widhalm K et al (2019) Healthy eating determinants and dietary patterns in European adolescents: the HELENA study. Child Adolesc Obes 2:18–39. 10.1080/2574254X.2019.1615361

[66] Bailey RL (2021) Overview of dietary assessment methods for measuring intakes of foods, beverages, and dietary supplements in research studies. Curr Opin Biotechnol 70:91–96. 10.1016/j.copbio.2021.02.007 33714006 10.1016/j.copbio.2021.02.007 PMC8338737

[67] Martínez ME, Marshall JR, Sechrest L (1998) Invited commentary: factor analysis and the search for objectivity. Am J Epidemiol 148:17–19. 10.1093/oxfordjournals.aje.a009552 9663398 10.1093/oxfordjournals.aje.a009552

[68] Michels KB, Schulze MB (2005) Can dietary patterns help us detect diet-disease associations? Nutr Res Rev 18:241–248. 10.1079/NRR2005107 19079908 10.1079/NRR2005107

