# Supplementary material for: Dietary patterns among children and adolescents in Germany consuming vegetarian, vegan or omnivore diets: results of the VeChi Youth Study
Source: Eur J Nutr. 2024 Sep 23;63(8):3161–74. doi: 10.1007/s00394-024-03497-6 (PMC11519145; doi:10.1007/s00394-024-03497-6)
Supplement: Supplementary file 1 — Supplementary Material 1 [file 394_2024_3497_MOESM1_ESM.docx]

**
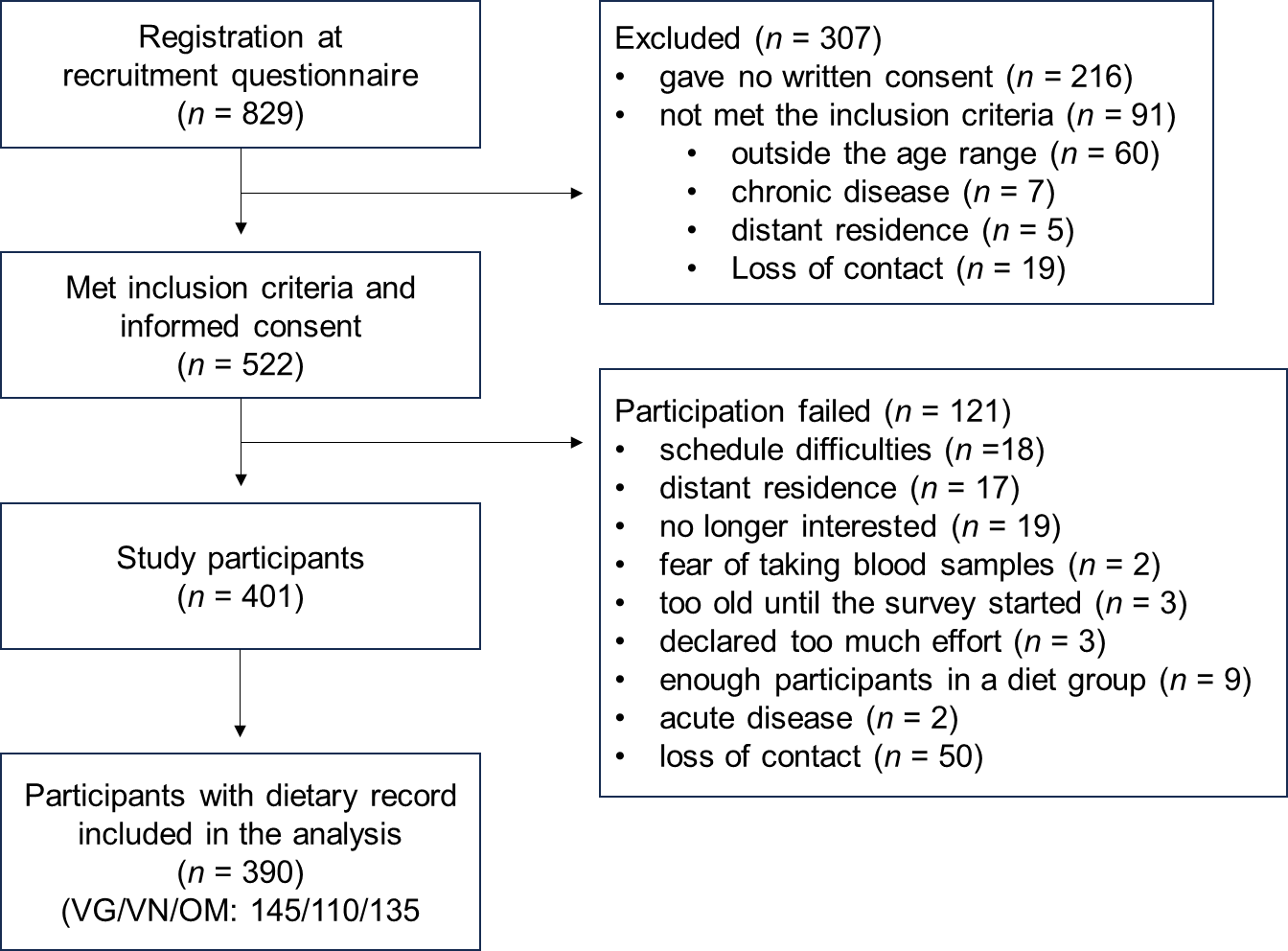
**

**Supplementary Figure 1:** Flow chart of recruitment of vegetarian (VG), vegan (VN), and omnivore (OM) children and adolescents (6-18 years old) in the VeChi Youth Study

**Supplementary Table 1**: Food group description

| Food group | Description |
| --- | --- |
| Vegetables | Fresh, frozen, canned and dried vegetables |
| Fruits | Fresh, frozen, canned and dried fruits |
| Whole grains ^1^ | Wholemeal flours, cereals, breads, breakfast cereals, muesli mixes, rice, bulgur, couscous, doughs, pasta |
| Refined grains | Refined flours, cereals, breads, breakfast cereals, muesli mixes, rice, bulgur, couscous, doughs, pasta |
| Potatoes | Potatoes, French fries, croquettes, potato dumplings, mashed potatoes |
| Legumes ^2^ | Beans, peas, lentils, lupines, soybeans (also as flour) |
| Nuts/seeds | Nuts (also nut butter, nut puree) and seeds (e.g., sesame seeds, sesame puree) |
| Plant oils/fats | Plant oils/fats, margarine |
| Animal fats | Butter, lard |
| Meat/sausage & fish | Meat (incl. poultry), sausage, ham, meat products, e.g., meat salad, cordon bleu, chicken wings, fish, fish products (e.g., fish sticks, herring salad), seafood |
| Eggs | Hen’s egg, scrambled egg, fried egg, egg salad, omelet |
| Fermented dairy products | Yogurt, kefir, buttermilk, creme fraiche, cream cheese, quark, semi-hard cheese, soft cheese, processed cheese |
| Unfermented dairy products | Milk, cream, condensed milk, milk powder |
| Plant based spreads | Plant based spreads based on legumes, vegetables, nuts or avocado |
| Meat alternatives | Vegetarian or vegan meat/sausage, patties, tofu, textured vegetable protein, soy cutlets |
| Dairy alternatives | Plant alternatives for milk, yogurt, cottage cheese and cheese |
| Sweet pastries | Sweet breads (e.g., raisin bread, chocolate croissant), pastries, cakes, pies, waffles |
| Sweets & snacks | Sugar, syrups, thick juices, jams, jellies, nut nougat creams, candies,chocolate, bars, ice cream (also vegan ice cream), snacks (e.g., chips, salt sticks, crackers) |
| Convenience Food | E.g., frozen pizza, canned soups, ready-made sauces (e.g., ketchup, dressing, pesto, mayonnaise), snack foods (e.g., burgers, kebabs) |
| Water | Drinking water, mineral water |
| Sugar sweetened beverages | Fruit juice drinks, fruit nectars, lemonades, cola drinks, iced tea, malt beer, instant beverage powder |
| Juices | Fruit and vegetable juices, juice spritzers, smoothies, squeezes |

Dry products or powders were considered with the respective water component.

^1^ Calculated according to HEALTHGRAIN definition (≥30% whole grains in total product and more whole grains than refined

grains).

^2^ weights were converted to consumption weights

**Supplementary Table 2**: Season and number of weekdays on which the 3-day weighed dietary records were completed by VeChi Youth Study participants (n = 390, 6-18 years old) stratified by diet group

|  | Diet Group | | |
| --- | --- | --- | --- |
|  | Vegetarian (*n* = 145) | Vegan  (*n* = 110) | Omnivore (*n* = 135) |
| Season |  |  |  |
| Winter | 31.3 | 40.0 | 34.8 |
| Spring | 13.6 | 15.5 | 21.2 |
| Summer | 14.2 | 14.5 | 24.2 |
| Autumn | 40.9 | 30.0 | 19.8 |
| Number of weekdays |  |  |  |
| 1 weekday, 2 weekend days | 17.2 | 10.9 | 14.8 |
| 2 weekdays, 1 weekend day | 55.2 | 64.5 | 57.8 |
| only weekdays (no weekend) | 27.6 | 24.6 | 27.4 |

Values are %

**Supplementary Table 3**: Factor loadings for the dietary patterns identified by principal components analysis among vegetarian children and adolescents in the German VeChi Youth Study (n = 145)

|  | Dietary pattern | | |
| --- | --- | --- | --- |
| Food group | Animal foods | Vegetables and fruits | Meat alternatives  and potatoes |
| Unfermented dairy products | **0.69** |  |  |
| Fermented dairy products | **0.60** |  |  |
| Animal fats | **0.54** | 0.26 |  |
| Eggs | **0.46** | -0.28 | 0.38 |
| Dairy alternatives | **-0.66** | 0.23 |  |
| Meat alternatives | **-0.52** |  | **0.46** |
| Legumes | **-0.40** | 0.34 |  |
| Vegetables |  | **0.58** | **0.48** |
| Fruits | -0.22 | **0.54** |  |
| Convenience Food |  | **-0.59** |  |
| Sugar sweetened beverages |  | **-0.51** |  |
| Potatoes |  | -0.29 | **0.65** |
| Juices |  |  | **0.42** |
| Sweet pastries |  |  | **-0.55** |
| Water | 0.23 | 0.35 |  |
| Sweets & snacks |  | -0.38 |  |
| Whole grains |  | 0.37 |  |
| Nuts/seeds |  | 0.38 | 0.33 |
| Plant based spreads | -0.38 |  |  |
| Plant oils/fats | -0.39 |  | 0.33 |
| Refined grains |  |  |  |
| Variance explained (%) | 13.0 | 10.7 | 9.0 |

Factor loadings ≥|0.4| are in in bold, factor loadings <|0.2| are not shown

**Supplementary Table 4:** Factor loadings for the dietary patterns identified by principal components analysis among vegan children and adolescents in the German VeChi Youth Study (n=110)

|  | Dietary pattern | | | |
| --- | --- | --- | --- | --- |
| Food group | Vegetables and legumes | Refined carbohydrates | Meat alternatives and juices | Fruits and convenience food |
| Vegetables | **0.81** | -0.22 |  |  |
| Potatoes | **0.68** |  |  |  |
| Legumes | **0.57** |  | -0.30 |  |
| Water | **0.54** |  |  | 0.36 |
| Sweets & snacks |  | **0.64** | -0.22 |  |
| Refined grains |  | **0.64** | 0.27 |  |
| Whole grains |  | **-0.66** | -0.27 |  |
| Dairy alternatives | -0.33 | **-0.53** |  | 0.21 |
| Meat alternatives |  |  | **0.76** |  |
| Juices |  |  | **0.56** |  |
| Nuts/seeds |  | -0.30 | **-0.59** |  |
| Fruits | 0.26 |  | -0.21 | **0.62** |
| Convenience Food |  | 0.24 |  | **0.56** |
| Sweet pastries |  |  |  | **-0.55** |
| Plant oils/fats | -0.24 |  | 0.21 | **-0.55** |
| Plant based spreads |  |  | 0.26 | 0.30 |
| Sugar sweetened beverages |  | 0.25 |  |  |
| Variance explained (%) | 12.3 | 11.2 | 10.1 | 9.7 |

Factor loadings ≥|0.4| are in in bold, factor loadings <|0.2| are not shown

**Supplementary Table 5:** Factor loadings for the dietary patterns identified by principal components analysis among omnivore children and adolescents in the German VeChi Youth Study (n=135)

|  | | Dietary pattern | | | | |
| --- | --- | --- | --- | --- | --- | --- |
| Food group | Flexitarian | | Vegetables and fruits | Dairy products | Meat and convenience food | Refined grains and juices |
| Whole grains | **0.73** | |  |  |  | -0.32 |
| Plant based spreads | **0.66** | |  |  |  |  |
| Dairy alternatives | **0.65** | |  | -0.30 |  |  |
| Meat alternatives | **0.43** | |  | -0.27 | -0.32 | 0.22 |
| Sweets & snacks | **-0.48** | |  | -0.29 | -0.24 | -0.33 |
| Vegetables |  | | **0.70** | -0.31 |  |  |
| Water |  | | **0.58** | -0.25 | 0.37 | -0.25 |
| Fruits |  | | **0.41** | -0.30 |  |  |
| Sweet pastries |  | | **-0.49** |  |  |  |
| Sugar sweetened beverages |  | | **-0.48** |  | 0.21 |  |
| Animal fats |  | |  | **0.74** | -0.22 |  |
| Unfermented dairy products |  | |  | **0.52** |  |  |
| Plant oils/fats |  | |  | **-0.47** | -0.23 |  |
| Legumes |  | | 0.31 | **-0.47** |  |  |
| Meat/sausage & fish | -0.22 | |  |  | **0.57** | 0.26 |
| Convenience Food |  | | -0.39 | -0.24 | **0.49** |  |
| Potatoes |  | |  |  | **-0.63** |  |
| Refined grains | -0.25 | | 0.37 |  |  | **0.63** |
| Juices |  | |  |  |  | **0.43** |
| Fermented dairy products |  | |  |  |  | **-0.60** |
| Eggs |  | | 0.31 | 0.20 | 0.22 | -0.21 |
| Nuts/seeds |  | |  |  | -0.34 | -0.31 |
| Variance explained (%) | 9.5 | | 9.5 | 8.9 | 7.7 | 7.4 |

Factor loadings ≥|0.4| are in in bold, factor loadings <|0.2| are not shown
